# Neonatal presentation of COG6‐CDG with prominent skin phenotype

**DOI:** 10.1002/jmd2.12154

**Published:** 2020-08-07

**Authors:** Katalin Komlosi, Selina Gläser, Julia Kopp, Alrun Hotz, Svenja Alter, Andreas D. Zimmer, Carmela Beger, Stefan Heinzel, Christoph Schmidt, Judith Fischer

**Affiliations:** ^1^ Institute of Human Genetics, Medical Center University of Freiburg, Faculty of Medicine, University of Freiburg Freiburg Germany; ^2^ Human Genetics Praxis Krone Laboratory Bielefeld Germany; ^3^ Neonatology Unit, Department of Pediatrics Children's Center Bethel, Evangelical Hospital Bethel Bielefeld Germany; ^4^ Genetikum®, Center for Human Genetics Neu‐Ulm Germany

**Keywords:** CDG, COG6, genodermatoses, restrictive dermopathy, WES

## Abstract

Many of the genetic childhood disorders leading to death in the perinatal period follow autosomal recessive inheritance and bear specific challenges for genetic counseling and prenatal diagnostics. Often, affected children die before a genetic diagnosis can be established, thereby precluding targeted carrier testing in parents and prenatal or preimplantation genetic diagnosis in further pregnancies. The clinical phenotype of congenital disorders of glycosylation (CDG) is very heterogeneous and ranges from relatively mild symptoms to severe multisystem dysfunction and even a fatal course. A very rare subtype, COG6‐CDG, is caused by deficiency of subunit 6 of the conserved oligomeric Golgi complex and is usually characterized by growth retardation, developmental delay, microcephaly, liver and gastrointestinal disease, joint contractures and episodic fever. It has been proposed that a distinctive feature of COG6‐CDG can be ectodermal signs such as hypohidrosis/hyperthermia, hyperkeratosis and tooth anomalies. In a Greek family, who had lost two children in the neonatal period, with prominent skin features initially resembling restrictive dermopathy, severe arthrogryposis, respiratory insufficiency and a rapid fatal course trio whole‐exome sequencing revealed the homozygous nonsense mutation c.511C>T, p.(Arg171*) in the *COG6* gene. Skin manifestations such as dry skin and hyperkeratosis have been reported in only five out of the 21 reported COG6‐CDG cases so far, including two patients with the c.511C>T variant in *COG6* but with milder ectodermal symptoms. Our case adds to the phenotypic spectrum of COG6‐CDG with prominent ectodermal manifestations at birth and underlines the importance of considering CDG among the possible causes for congenital syndromic genodermatoses.

**SYNOPSIS**

Our case adds to the severe neonatal presentation of COG6‐CDG with prominent skin manifestations at birth and emphasizes the importance of considering the congenital disorders of glycosylation among the possible causes for congenital syndromic genodermatoses.

## 1 INTRODUCTION

Many of the metabolic diseases leading to death in the perinatal period follow autosomal recessive inheritance and bear specific challenges for genetic counseling and prenatal diagnostics in future pregnancies. Carrier parents are clinically unaffected, and diseases are rare but have recurrence risks of 25% in the same family. Often, affected children die before a genetic diagnosis can be established, precluding targeted carrier testing in parents and prenatal or preimplantation genetic diagnosis in further pregnancies.

Restrictive dermopathy (RD, MIM: #275210) is a rare, lethal genodermatosis with manifestations easily recognizable at birth: tense, vulnerable and translucent skin, superficial erosions, joint contractures, reduced motoricity and a typical facies with a small pinched nose, mouth fixed in an o‐position, low‐set ears, micrognathia, and respiratory insufficiency.^1^ Prenatal signs include intrauterine growth retardation, reduced fetal movements, polyhydramnios, and premature rupture of the amniotic membrane. Most infants show a fatal course and die within the first week of life.^1^, ^2^, ^3^

Congenital disorders of glycosylation (CDG) are due to defects in the glycosylation of glycoproteins or glycolipids. The clinical phenotype of CDG ranges from mild symptoms to severe multisystem dysfunction and even a fatal course^4^ including forms also classified under syndromic ichthyosis.^5^, ^6^ Seven known CDGs are deficiencies in the conserved oligomeric Golgi (COG) complex (COG1, 2, 4, 5, 6, 7, and 8)^7^, ^8^, ^9^ that controls Golgi trafficking, processing, and sorting.^10^ COG6‐CDG (MIM: #614576) is caused by deficiency of subunit 6 of COG, and is characterized by growth retardation, global developmental delay, muscular hypotonia, microcephaly, liver and gastrointestinal disease, thrombocytopenia, recurrent infections, episodic fever, congenital heart defects, generalized joint contractures, and early lethality.^11^, ^12^, ^13^, ^14^ It has been proposed that a distinctive feature of COG6‐CDG compared to other CDG can be additional ectodermal signs comprising hypohidrosis/hyperthermia, thickened skin, hyperkeratosis, dry skin, and tooth anomalies.^11^, ^12^, ^13^, ^14^, ^15^

Here we report two additional cases of COG6‐CDG with the previously described ultra‐rare homozygous nonsense mutation c.511C>T, p.(Arg171*) in the *COG6* gene^12^ presenting with prominent skin features at birth resembling restrictive dermopathy and a fatal course with respiratory insufficiency, thrombocytopenia, and hepatosplenomegaly.

## 2 CASE SUMMARY 

**FIGURE 1:**
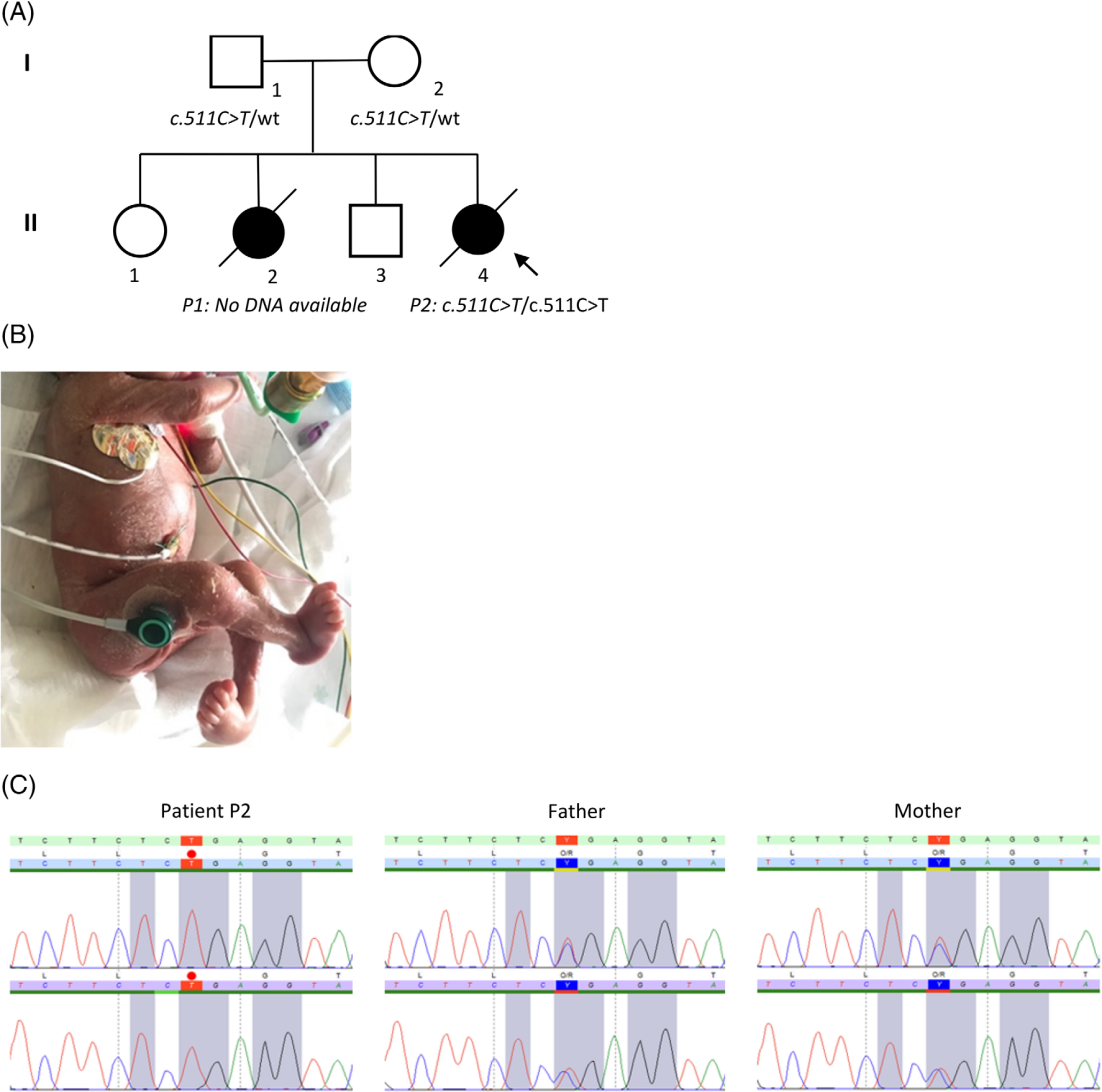
A. Pedigree of the family indicating heterozygosity or homozygosity for the c.511C>T variant in the *COG6* gene. The arrow indicates the proband. B. Photo of patient P2 revealing the skin phenotype and joint contractures. C. Electropherogram of the Sanger sequencing showing the homozygous nonsense mutation c.511C>T, p.(Arg171*) in the *COG6* gene in the proband confirming the NGS results; in order of the sequence: proband P2, the red bar indicates the homozygous base exchange; father and mother, respectively, blue bars indicate the heterozygous state of the sequence variant. The confirmation of the NGS results and the segregation analysis was performed by Sanger sequencing on an ABI 3500 DNA Sequencer

The index patient (P2, Figure 1A) was the fourth child of healthy nonconsanguineous parents from Greece. The first‐born child was a healthy 9‐year‐old girl and the third child was a healthy 4‐year‐old boy at the time of diagnostic testing of the proband, the second child (P1, Figure 1A) died at the age of 15 days due to respiratory insufficiency and had shown clinical signs very similar to those of the fourth child (P2). Unfortunately, no medical records of the second child (P1) were available and the parents reported that no definitive diagnosis had been achieved. According to the descriptions of the parents the female child was born prematurely, showed a very dry and tight skin, generalized joint contractures, and had to be ventilated immediately after birth. After extubation, the girl died on day 15. Pregnancy with the fourth child had been complicated around gestational week 25 by oligohydramnios, and on prenatal ultrasound moderate cardiomegaly, lung hypoplasia, intrauterine growth retardation, hyperechogenic liver and clubfoot of the fetus were observed. Prenatal FISH analysis of amniotic fluid excluded trisomy 13, 18, and 21. In gestational week 29 a normal amount of amniotic fluid for gestational age was recorded. The girl was born prematurely after cesarean section because of fetal distress at 30 + 2 gestational weeks with a birth weight of 975 g (10th percentile), birth length of 35 cm (3rd‐10th percentile) and a head circumference of 26.5 cm (10th‐25th percentile). Apgar score was 1/5/7. The child showed severe muscular hypotonia and had no spontaneous breathing and was intubated and ventilated, with postnatal adaptation complicated by respiratory insufficiency due to lung hypoplasia. She showed signs of arthrogryposis with talipes equinovarus, generalized joint contractures and thoracolumbal scoliosis. She had a small pinched nose, dysplastic ears and incomplete eye closure. The most striking feature was her very dry, tight and rigid skin with skin erosions, hyperkeratosis, scaling (Figure 1B), and nearly absent eyelashes and eyebrows. A skin biopsy sample was taken at the Neonatology Unit and examined at an external laboratory showing unspecific changes typical for an immature development of the epidermis and dermis. Many of the clinical features at birth resembled restrictive dermopathy and the histology was described to be compatible with secondary changes of RD; however, no specific changes for RD were noted. Abdominal ultrasound detected hepatosplenomegaly and echocardiography demonstrated a persistent foramen ovale. Brain ultrasound was normal. Laboratory investigations revealed hyponatremia and thrombocytopenia (49 G/l). In spite of continuous ventilation the patient's clinical state worsened, and on the fourth day of life, she developed a global respiratory insufficiency and expired.

## 3 RESULTS

According to the initial clinical suspicion of restrictive dermopathy Sanger sequencing of the two known causative genes *LMNA* and *ZMPSTE24* was carried out but detected no pathogenic alterations. For further analysis postmortem trio whole‐exome sequencing (Agilent SureSelect XT Human All Exon Kit V6, Agilent Technologies, Santa Clara, California) was performed and revealed the homozygous nonsense mutation c.511C>T, p.(Arg171*) in the *COG6* gene in the index patient (P2). Both parents were heterozygous carriers. The mutation was validated by Sanger sequencing (Figure 1C). This variant was already described by Rymen et al^12^ and is listed in ClinVar (NM_001145079.1(COG6): c.511C>T, (p.Arg171*), https://www.ncbi.nlm.nih.gov/clinvar/variation/493007) and HGMD (CM1512209) as pathogenic. Following standard filtering only two additional homozygous variants remained in the index patient (Supplementary Table 1.): a homozygous missense variant of uncertain significance in the *NOXA1* gene (MIM *611255) and a homozygous missense variant of uncertain significance in the *NOTCH1* gene (MIM *190198). No further causative or possibly causative variants were identified in the trio analysis that would explain the striking phenotype in the index patient. Biallelic pathogenic variants in the *COG6* gene (MIM *606977) are responsible for COG6‐CDG (MIM #614576).

## 4 DISCUSSION

We present two siblings with a lethal neonatal phenotype of COG6‐CDG whose striking skin features initially raised the suspicion of an underlying restrictive dermopathy. Since the symptoms of the disease were already present prenatally and involved multiple organ systems including severe arthrogryposis, hepatosplenomegaly, thrombocytopenia, and respiratory insufficiency and showed a rapid fatal course, the overall clinical picture pointed toward a glycosylation disorder. Dry skin, tight and rigid skin with hyperkeratosis and scaling at birth represents the clinical phenotype of heterogeneous diseases ranging from nonsyndromic genodermatosis to fatal multiorgan disorders including inborn errors of metabolism. Restrictive dermopathy manifestations include a tense, vulnerable and translucent skin with prominent vessels, superficial erosions, joint contractures, reduced motoricity, a typical facies, and respiratory insufficiency.^1^ Although both of the siblings we present here showed tightly adherent skin and generalized joint contractures, they also presented with immediate respiratory insufficiency, hepatosplenomegaly, and thrombocytopenia. During the short life span of the index patient (P2) only sequencing of *ZMPSTE24* and *LMNA* known to be causative for restrictive dermopathy was performed and detected no pathogenic alterations. With the aim of providing the parents exact recurrence risks and the opportunity to obtain prenatal diagnostics in future pregnancies trio whole‐exome sequencing was performed postmortem and revealed the homozygous nonsense mutation c.511C>T, p.(Arg171*) in the *COG6* gene in the proband (P2, Figure 1C) leading to the diagnosis of COG6‐CDG. Both parents were heterozygous carriers. No postmortem sample was available from the first deceased child (P1, Figure 1A); however, the phenotypic description of the parents showed striking similarity to the manifestations of the second affected child. Unfortunately, the functional relevance of the homozygous variant could not be supported by isoelectric focusing of serum transferrin in our patients.

Although no consanguinity is known between the parents of our patients, the presence of a homozygous ultra‐rare variant hypothesizes some degree of consanguinity and increases the chances of a second unrelated genetic error that may explain the prominent skin phenotype. Following standard filtering only three homozygous variants remained in the index patient (Supplementary Table 1): the previously described pathogenic missense variant in *COG6*, and two variants with uncertain significance: a homozygous variant in the *NOXA1* gene (MIM *611255) that is not yet associated with a disease entity and a homozygous missense variant in the *NOTCH1* gene (MIM *190198) causative of autosomal dominant Aortic valve disease (MIM #109730) and Adams‐Oliver syndrome Type 5 (MIM #616028). We do not think that the two Class 3 variants exerted a significant contribution to the skin phenotype. We were not able to identify additional variants that would have possibly contributed to the skin phenotype, nor were we able to identify any pathogenic variants in genes associated with the most closely related HPO terms of the skin phenotype seen in our patients (for the gene list see Supplementary Table 2). The two patients reported so far with the c.511C>T variant in *COG6*_12_ and our patients originate from the same geographical area of South‐Eastern Europe (Bulgaria, Turkey and Greece), thus the variant could also possibly represent a local founder mutation. The gnomAD database lists eight heterozygous carriers, two from the Askhenazi Jewish, and six from the European (non‐Finnish) population. Comparative haplotype analysis of other families carrying the same mutation will be the approach to resolve this possibility.

Skin abnormalities are described in about 20% of the different CDG forms^5^, ^6^, ^9^, ^16^, ^17^, ^18^ and include abnormal fat distribution, lipodystrophy, cutis marmorata,^19^ excessive skin wrinkling, orange peel skin,^17^ ichthyosis,^5^, ^6^ hyperkeratosis,^9^ increased skin laxity, hypo−/hyperpigmentation, tumoral calcinosis, aplasia cutis congenita,^18^ and hypohidrosis/hyperthermia.^15^, ^16^ Ichthyosis or ichthyosiform dry skin with variable neurologic and multiorgan involvement is particularly a feature of four types of CDG caused by mutations in the genes *MPDU1*, *DOLK*, *SRD5A3*, and *PIGL*.^16^, ^20^ It has been proposed that a distinctive feature of COG6‐CDG compared to other CDG can be additional ectodermal signs including hypohidrosis/hyperthermia, dry, orange peel skin, hyperkeratosis, and tooth anomalies.^11^, ^12^, ^15^ Reviewing the literature, however, abnormality of the skin is reported only in about one third of all COG‐CDG (13/48 cases) and in about one third of all COG6‐CDG (6/21) cases (References 11, 12, 20 and our patients). No case has been reported so far with the description of dry, tight and rigid skin at birth (Table 1). Applying the approach of Haijes et al^11^ to our patients to assess the relevance of phenotypic similarities (Supplementary Table 2), we found evidence of similar skin phenotype terms in a few other COG‐CDG cases: “thickened skin,” “epidermal thickening,” and “hyperkeratosis” is mentioned in 5/21 COG6‐CDG cases while “dry skin” in 4/21 COG6‐CDG cases and in 1/3 COG8‐CDG case but “tight skin” (lack of skin elasticity) only in our two patients (2/21).^11^, ^21^ Due to the scarce reports of the skin phenotype in most cases phenotypic specificity was very low for most skin related HPO terms, only for hypohidrosis did it reach 0.20. The biochemical mechanisms for the skin symptoms in COG‐CDG are not known yet and there are no reports of the dermatopathological changes in the few described patients. Unfortunately, there was no precise histopathological description in our patient available either. Skin histology is presented in a neonatal case of DOLK‐CDG with severe ichthyosis at birth and showed lipid droplet accumulation in the stratum corneum and keratinocytes, suggestive of defective epidermal lipid metabolism. It was hypothesized that epidermal lipid metabolism may be dependent on normal glycosylation and could be perturbed in various CDG defects.^22^ We chose the HPO term “tight skin” (lack of skin elasticity) to assess the pathophysiology in other diseases presenting with “tight skin” (gene ontology for 41 genes) in an attempt to identify additional cellular functions possibly accounting for the skin features (Supplementary Table 2). We were not able to assess a specific pattern apart from the overrepresentation of cell signaling among those gene functions. More COG6‐CDG patients with the precise description of ectodermal features will be needed to study the underlying pathophysiology systematically.

**TABLE 1:** Reported COG6‐CDG patients with skin abnormalities

	Rymen et al (P3)^12^	Rymen et al (P4.1)^12^	Rymen et al (P4.2)^12^	Rymen et al (P6.1)^12^	Shaheen et al^22^	Our patient
Age/sex	15 months/F	21 years/M	14 months/M	12 years/F	13 years/M	4 days/F
*COG6* variants	c.1238_1239insA, p.(Phe414Leufs*4) hom	c.1646G>T, p.(Gly594Val)/c.785A>G, p.(Tyr262Cys)	c.1646G>T, p.(Gly594Val)/c.785A>G, p.(Tyr262Cys)	c.511C>T, p.(Arg171*)/c.1746+2G>T	c.1167‐24A>G hom	c.511C>T, p.(Arg171*) hom
Skin	Hyperkeratosis	Hyperkeratosis of palms and soles	Dry skin	Orange peel skin	Palmoplantar hyperkeratosis	Tight, rigid, dry skin resembling restrictive dermopathy
Hypohidrosis/hyperthermia	NA	Yes	Yes	Yes	Yes	NA
Cause of death	Liver failure	–	Liver failure	–	–	Respiratory failure
Facial dysmorphism	Retrognathia, broad palpebral fissures	Wide mouth with thin lips, prominent nose, slight epicanthus	Not described	Epicanthic folds, tubular nose, large mouth with gingiva hyperplasia	Flaring of the lateral eyebrows, broad nasal tip, full lips	Small nose, O‐shaped mouth
Growth retardation	Yes	No	No	Yes	NA	Yes
Microcephaly	Yes	Yes	Yes	Yes	No	Yes
Brain anomalies	Cerebral and cerebellar atrophy	NA	NA	Cortical atrophy	NA	US normal
Congenital heart defect	ASD, PDA	NA	NA	VSD	NA	PFO
Pulmonary hypoplasia	No	No	No	No	No	Yes
Gastrointestinal tract	Chronic diarrhea	Normal	NA	Chronic diarrhea	NA	NA
Liver and biliary tract	Hepatosplenomegaly	Splenomegaly	Hepatosplenomegaly, cholestasis, liver failure	Hepatosplenomegaly, cholestasis, cirrhosis	NA	Hepatosplenomegaly
Skeletal anomalies	Postaxial polydactyly, metopic synostosis	No	No	Hypermobility of joints, scoliosis	NA	Clubfoot, joint contractures, scoliosis
Hematological abnormalities	Thrombocytopenia	Thrombocytopenia	NA	Mild pancytopenia	NA	Thrombocytopenia

Abbreviations: ASD, atrial septal defect; F, female; M, male; NA, not assessed; PDA, patent ductus arteriosus; PFO, patent foramen ovale; US, ultrasound; VSD, ventricular septal defect.

Due to the limited number of COG6‐CDG patients reported to date, no clear genotype‐phenotype correlation has been established. Only the deep intronic splice site mutation (c.1167‐24A>G) is clearly correlated with Shaheen syndrome (OMIM #615328,^23^). The nonsense mutation *COG6*: c.511C>T, p.(Arg171*) detected in our patient was previously described by Rymen et al: patient P1, who was homozygous for the variant, presented with hypohidrosis and hyperthermia but no other skin abnormality, patient P6.1, who was compound heterozygous, had orange peel skin and hypohidrosis (Reference 12, Table 1). In contrast, the most striking feature in our patients was the dry, tight and rigid skin. Hypohidrosis and hyperthermia could not be assessed during the short life span of our patient (P2) placed into an incubator immediately after birth, nor was data available from the first affected child (P1, Figure 1A). Clinical features common to all three patients confirmed to carry the c.511C>T, p.(Arg171*) variant were facial dysmorphism, growth retardation, microcephaly, congenital heart defect, hepatosplenomegaly, skeletal anomalies, and thrombocytopenia. Patients homozygous for the c.511C>T variant additionally showed multiple joint manifestations (clubfoot, opposed thumbs, hip dysplasia, scoliosis). The course of the disease was similarly severe and fatal with immediate intubation and ventilation after birth and death after stopping ventilation.

In the pregnancy with our index patient (P2, Figure 1A), oligohydramnios, moderate cardiomegaly, lung hypoplasia, intrauterine growth retardation, hyperechogenic liver and clubfoot of the fetus were observed around gestational week 25. However, in gestational week 29 a normal amount of amniotic fluid was recorded, so there was no evidence of a longstanding oligohydramnios. Nevertheless, a potential role of the oligohydramnios in the skin features of our patient cannot be ruled out. However, in contrast with the skin observations of Hall^24^ examining the relationship of longstanding oligohydramnios to arthrogryposis and the secondary and/or tertiary effect on fetal skin, wrinkled skin was only observed on the upper arms in our patient (P2), but no extra skin or skin creases were present elsewhere. She rather presented with tight skin. We do not think, therefore, that the skin features in our patient only developed as a secondary effect of the oligohydramnios but rather believe that the rigid and tight skin may represent the severe end of the spectrum of skin manifestations that have been observed in other COG‐CDG cases so far.

The family we describe here adds to the severe neonatal presentation of COG6‐CDG with prominent skin manifestations at birth and emphasizes the importance of considering the congenital disorders of glycosylation among the possible diagnoses for congenital genodermatoses.

## 5 CONCLUSION

Our case adds to the broad phenotypic spectrum of COG6‐CDG and underlines the value of trio exome sequencing in children with severe disease course and early perinatal death. Reaching a molecular genetic diagnosis in such fatal cases is, however, a requirement for targeted carrier testing in parents and prenatal or preimplantation genetic diagnosis in further pregnancies.

## CONFLICT OF INTEREST

K. K., S. G., J. K., A. H., S. A., A. D. Z., C. B., S. H., C. S., and J. F. declare that they have no conflict of interest.

## AUTHOR CONTRIBUTIONS

Judith Fischer conceived the case report. Stefan Heinzel performed the clinical diagnosis and treatment of the neonate and initiated the genetic diagnostics and Carmela Beger carried out the genetic counseling and further genetic diagnostics of the proband and her parents; Julia Kopp, Alrun Hotz, Svenja Alter and Judith Fischer carried out the molecular analyses and reviewed the molecular genetic results; Andreas D. Zimmer and Christoph Schmidt performed the bioinformatic analyses; Katalin Komlosi and Selina Gläser planned the case report, interpreted the results, researched the literature and prepared the manuscript. Stefan Heinzel, Carmela Beger, Julia Kopp, Alrun Hotz, Svenja Alter, Andreas D. Zimmer and Christoph Schmidt edited and reviewed the manuscript, Judith Fischer contributed substantially to the conception, design, and critical revision of the work for important intellectual content. Katalin Komlosi and Selina Gläser drafted the paper and coordinated writing of the manuscript. All authors discussed, read, and approved the manuscript. All authors approve the version to be published and agree to be accountable for all aspects of the work in ensuring that questions related to the accuracy or integrity of any part of the work are appropriately investigated and resolved.

## INFORMED CONSENT

All procedures followed were in accordance with the ethical standards of the responsible committee on human experimentation (institutional and national) and with the Helsinki Declaration of 1975, as revised in 2000 (5). Informed consent was obtained from all patients for being included in the study. This study was carried out through routine diagnostic activity; formal ethics review was therefore not requested by our institutional ethical committee.

Additional informed consent was obtained from all patients for which identifying information is included in this article.

## ANIMAL RIGHTS

This article does not contain any studies with human or animal subjects performed by the any of the authors.

## Supporting information


**Table S1** Supporting information

**Table S2** Supporting information

## Data Availability

All relevant data generated or analyzed during this study are included in this published article. The complete datasets used and/or analyzed during the current study are available from the corresponding author upon request.
